# MSALigMap—A Tool for Mapping Active-Site Amino Acids in PDB Structures onto Known and Novel Unannotated Homologous Sequences with Similar Function

**DOI:** 10.3390/life12122082

**Published:** 2022-12-12

**Authors:** Sameer Hassan, Sameena Haleemath Sameer, Mats Töpel, Henrik Aronsson

**Affiliations:** 1 Department of Biological and Environmental Sciences, University of Gothenburg, P.O. Box 461, 405 30 Gothenburg, Sweden; 2 OlsAro Crop Biotech AB, Erik Dahlbergsgatan 11A, 411 26 Gothenburg, Sweden; 3 Department of Computer and Systems Sciences, Stockholm University, Kista, 106 91 Stockholm, Sweden; 4 Department of Marine Sciences, University of Gothenburg, P.O. Box 461, 405 30 Gothenburg, Sweden; 5 IVL Swedish Environmental Research Institute, Aschebergsgatan 44, 411 33 Gothenburg, Sweden

**Keywords:** binding, DNA, mapping, multiple sequence alignment, ligand, peptide, protein, python

## Abstract

MSALigMap (Multiple Sequence Alignment Ligand Mapping) is a tool for mapping active-site amino-acid residues that bind selected ligands on to target protein sequences of interest. Users can also provide novel sequences (unavailable in public databases) for analysis. MSALigMap is written in Python. There are several tools and servers available for comparing and mapping active-site amino-acid residues among protein structures. However, there has not previously been a tool for mapping ligand binding amino-acid residues onto protein sequences of interest. Using MSALigMap, users can compare multiple protein sequences, such as those from different organisms or clinical strains, with sequences of proteins with crystal structures in PDB that are bound with the ligand/drug and DNA of interest. This allows users to easily map the binding residues and to predict the consequences of different mutations observed in the binding site. The MSALigMap server can be accessed at https://albiorix.bioenv.gu.se/MSALigMap/HomePage.py.

## 1. Introduction

Recently, genome and mRNA sequencing revealed the possibility to identify a large number of genes and transcripts [1,2]. As sequencing data have become more accessible, the primary interest has shifted from sequencing to the annotation of variations to explain protein characteristics. Many of these genes or protein sequences are homologous to annotated sequences in other genomes, and many are identified as novel genes with unknown function. Genes with unknown functions still play important roles in determining cell phenotypes [3]. While a large number of genomes are sequenced at an ever-increasing pace, predicting the function of the genes encoded in these genomes has emerged as a new area of research. Annotating the function of protein sequences remains one of the most important issues in understanding the molecular mechanisms of life [4] and has great implications in biology and pharmaceutical research [5,6]. The computational methods often used for predicting protein function are structure- and sequence-based methods [7], and multiple sequence alignment is one of the key steps in many bioinformatics analyses. Hence, protein binding characterization and quantitative comparison have long been of interest to the scientific community [8], and sequence alignment is an important component of such analyses.

Transcription factors are important for genetic regulation in all organisms and recognize sequence-specific regions in DNA called transcription factor binding sites (TFBSs). Through this type of interaction, transcription factors regulate (induce or repress) the expression of their target genes [9]. Annotating TFBSs is an important step in genome annotation. Many structure- and sequence-based methods have been developed for predicting TFBSs [10,11]. The recent increase in the number of experimentally resolved structures of protein–DNA complexes in the PDB database can help to annotate the DNA binding sites of novel transcription factor proteins identified in whole-genome sequencing projects that are homologous to these structural complexes.

Identifying binding sites is essential to achieving an understanding of catalytic reactions and to the classification of enzyme proteins involved in various biological processes. The number of 3D protein structures deposited in RCSB Protein Data Bank (PDB) [12,13] has greatly increased over the years (currently, >190,000 protein structures) and provides an excellent opportunity to study the conservation of amino acids involved in ligand binding across different protein families, species, and strains. With the increasing number of protein sequences obtained in large sequencing projects being deposited into, among others, the NCBI [14], ENSEMBL [15], and DDBJ [16] databases, it becomes ever more important to combine and compile data in order to identify residues that are involved in, for example, ligand binding. One strategy for mapping ligand binding residues is based on comparing homologous 3D protein structures from PDB that are bound with the ligand of interest and whose function and binding site are already characterized. For this purpose, it is important that known active-site residues from the crystallized characterized 3D protein structures can be transferred to uncharacterized protein sequences in a simple manner in order to identify the functionally and catalytically important residues.

Many excellent tools have been developed for this purpose; however, they have the limitations that they can be only used for comparing individual sequences against structural data. FeatureMap3D, a web-based tool, allows protein features to be mapped onto protein structures separately for each sequence submitted [17]. Many structure-based tools, such as LigAlign [18] and GASS-WEB [19], are available for mapping binding pockets using protein structures. Recently, it was reported that XSuLT, a web-based server, can be used for sequence annotation using structural information [20]. However, this tool cannot be used for mapping the DNA binding sites of transcription factor proteins.

As protein sequences provide insights into protein function, the mapping of functionally important amino acids from three-dimensional complexes onto proteins with unknown function can assist in protein evolutionary analyses and protein design. This demands for tools that can be used to transfer annotated features from characterized protein sequences to novel sequences. Given the necessity of transferring binding-site information about ligands such as drugs, DNA, substrates, or cofactors for large uncharacterized sequences from homologous PDB 3D protein structures, we developed MSALigMap, a user-friendly Python-based tool that requires only two input files. This tool can be used to assist the user in mapping the binding residues onto homologous non-structure protein sequences based on the sequences of PDB 3D protein structures provided by the user. However, this tool can be used particularly when the sequence identity for the selected sequences with similar function is above the twilight zone, i.e., above 30% identity. The MSALigMap server can be accessed at https://albiorix.bioenv.gu.se/MSALigMap/HomePage.py.

## 2. Materials and Methods

MSALigMap is a web-based tool (https://albiorix.bioenv.gu.se/MSALigMap/HomePage.py) developed in Python (>3.0) for protein sequence alignment and the mapping of functionally important amino acids onto known and novel unannotated homologous sequences with similar function. The web interface was developed in HTML on the XAMPP server running on a Linux system. Python CGI programming was used for developing MSALigMap. This tool was not designed to generate alignment of its own; rather, the current version uses MAFFT [21] for multiple sequence alignment. The MSALigMap server depends on Biopython [22]. The SeqIO class module from Biopython is used for reading the sequence file input. All input and output functionalities are performed in standard Python. The major challenge of the server is the quality of multiple sequence alignment generated, which depends upon the sequences selected by the user for the analysis.

MSALigMap analyzes each of the sequences and structures in the provided sequence file. The definition of secondary structure of proteins (DSSP) of the structure is used for extracting 2D details from the 3D protein structures. PDBsum is used for extracting the ligand-binding- and DNA-binding-site information. The command line version of ClustalO is used for performing the multiple sequence alignment of the user-specified protein sequences.

**Figure 1:**
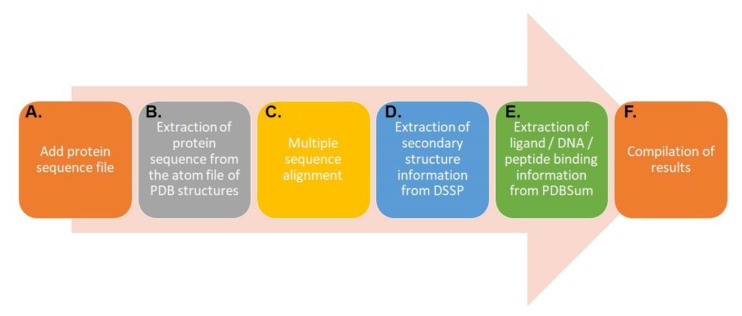
Flowchart for the basic workflow using MSALigMap: First, a protein sequence file of interest is identified (**A**) before the extraction of a protein sequence from the atom file of the PDB structures (**B**). These sequences are then aligned (**C**). Using DSSP, the secondary structure of the PDB structures is extracted (**D**). In this step, the ligand, DNA, or peptide binding information is extracted from PDBsum (**E**). This leads to the compilation of the results (**F**).

The basic workflow is illustrated in Figure 1. For mapping small-molecule binding sites, two input files are required: a sequence file in FASTA format and a PDB code with chain and ligand information, here exemplified with the PDB crystal structure of carbonyl reductase (3WXB:A) [23]. On the other hand, for protein–peptide binding-site analyses, the files required are a sequence file in FASTA format and the PDB code, which is provided here with the example from the protein sequence of the LRX crystal structure of the LRX protein in complex with the RALF peptide from Arabidopsis, i.e., 6QWN [24]. For mapping DNA binding sites, a sequence input file in FASTA format is required, where the sequence header of the structure has the PDB code and chain information, exemplified here with the Arabidopsis WRKY4 domain, AtWRKY4, in complex with DNA (2LEX) [25].

The identified PDB codes are searched in the PDB database and downloaded (Figure 1A). The protein sequence file of the protein structures is extracted from the PDB file for computational analyses (Figure 1B).

The protein sequences are extracted from the PDB atom file, and the non-structure protein sequences are aligned using the ClustalO tool, which is installed locally (Figure 1C). There are different multiple sequence alignment programs, and it is important that the user makes sure that the alignment is strongly homologous, i.e., at least 30% protein sequence identity. Several programs can be used; we used ClustalO. There is currently no limitation when it comes to the number of sequences to be used to run multiple sequence alignment; however, the higher the number is (e.g., 100 sequences), the more time is needed to run the calculation. Thus, there is no threshold for multiple sequence alignment, but a slower run is expected for higher numbers.

The DSSP protein secondary structure annotations of the PDB 3D protein structures are extracted from the MRS server [26] using the BeautifulSoup module in Python (Figure 1D). Thereafter, the binding-site analysis starts.

For protein–ligand and protein–peptide complexes, the ligand and peptide binding amino-acid residues whose interactions are classified as hydrogen-bonded or non-bonded are extracted using the LigPlot [27] output available on the PDBSum [28] database for the computational analysis. For protein–DNA complexes, the DNA-binding-site amino acids are identified using NucPlot [29] from the PDBSum database (Figure 1E).

The mapping of functionally important amino acids is then performed with MSALigMap (Figure 1F). MSALigMap analyzes each of the structures in the alignment of the features identified in the above steps. These data are transformed into an HTML-formatted file for displaying the annotated features as presented in Table 1. The mapped binding-site information on protein structures and sequences is displayed in alignment format for a better understanding of the identical and substituted amino acids identified in the sequences. Identical positions are colored in red, and substituted positions are colored in blue shades.

## 3. Results

**Figure 2:**
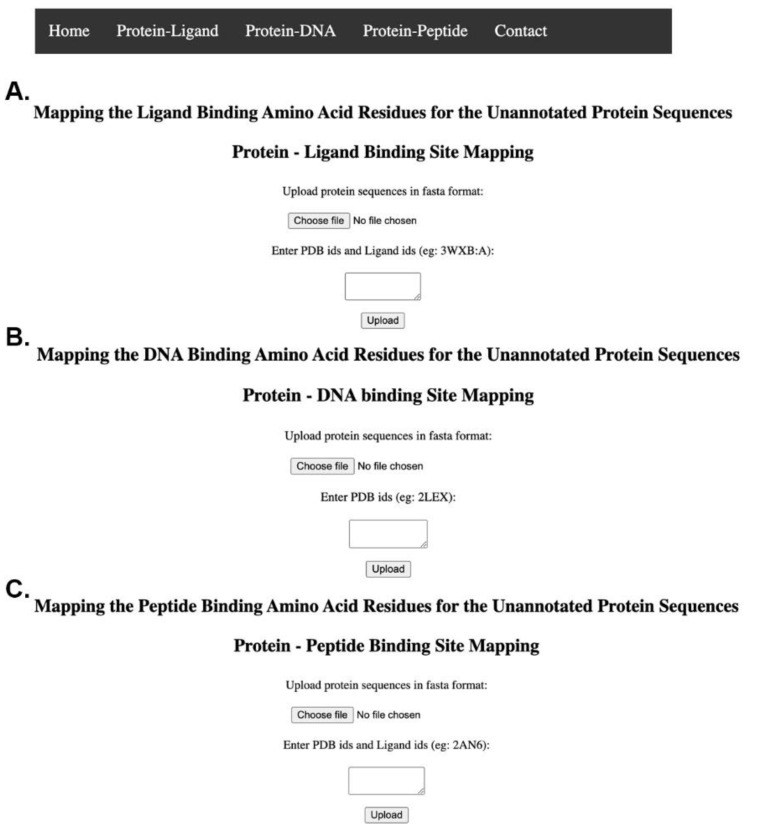
Screenshots of MSALigMap tool. The tool has options for analyzing interactions between protein–ligand complexes (**A**), protein–DNA complexes (**B**), and protein–peptide complexes (**C**).

MSALigMap is a web-based feature annotation tool for annotating functionally important amino acids that interact with ligands, peptides, and DNA molecules. The server is freely available at https://albiorix.bioenv.gu.se/MSALigMap/HomePage.py. The input sequences are submitted through the URL. The current version of the server has options for separately analyzing protein–ligand, protein–DNA, and protein–peptide complexes (Figure 2). The protein–ligand and protein–peptide programs require two inputs: (i) a multiFASTA unaligned sequence file, which can be uploaded as a file, and (ii) comma-separated PDB codes with chains (e.g., 3WXB:A, 3O26:A) [23,30], for DNA (e.g., 2LEX), and peptides (e.g., 6QWN) [24]. However, for protein–DNA complex analyses, a multiFASTA unaligned sequence file, which can be uploaded as a file, is required. The FASTA header of the sequences of protein structures should have a PDB code and chain information (e.g., 2LEX:A) [25].

It is essential that the sequence identifiers of the protein structures match the PDB codes and are identical in the sequence file and information in the text box for ligand information in protein–ligand analyses. If the provided PDB codes do not match the standard format of the PDB database, the program considers the sequence as a non-structure sequence. The server output consists of two sections: first, a formatted alignment of PDB sequences and non-structure sequences with color-coded information for secondary structures and binding-site amino acids; second, the sub-section of aligned binding-site amino acids that are color-coded based on positions that are identical (red) and substituted (blue) across all sequences.

### 3.1. MSALigMap Example: Protein–Ligand Analysis

**Figure 3:**
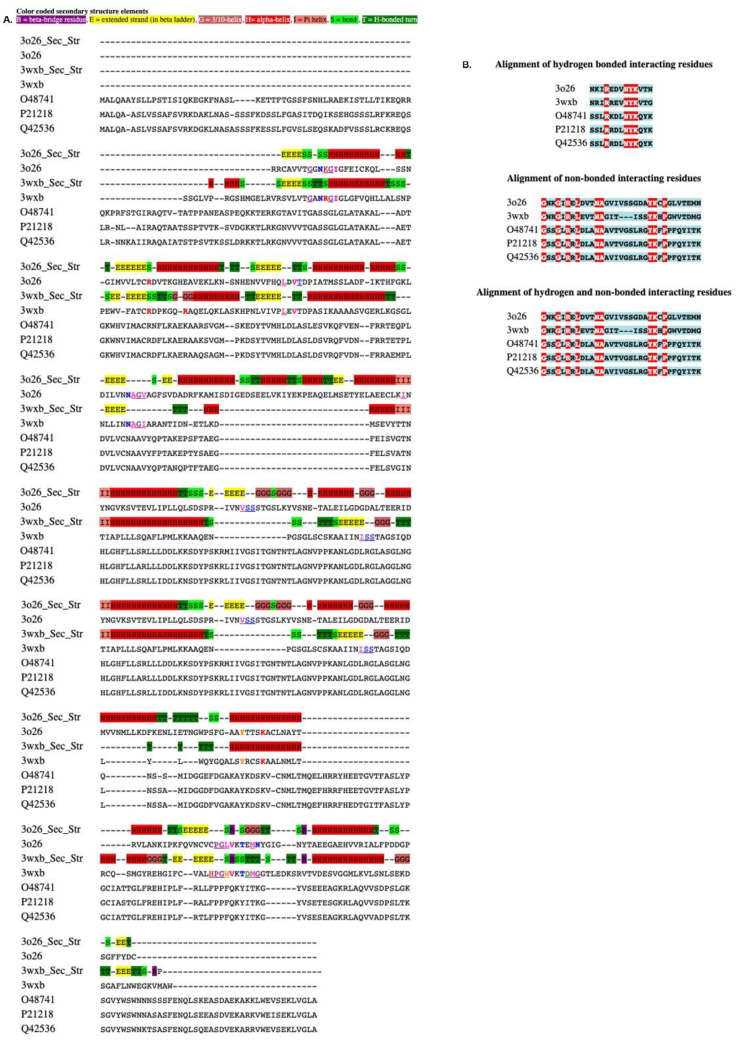
Multiple sequence alignment of NADPH:protochlorophyllide oxidoreductase (POR) is used as a representative example of a protein–ligand analysis: (**A**) alignment with color-coded secondary structures; (**B**) hydrogen-bonded and non-bonded interacting residues retrieved with the alignment. Conserved amino acids are shown in bold/are framed with a square. B, beta-bridge residue; E, extended strand (in beta ladder); G, 3/10 helix; H, hydrogen bond; I, Pi-helix; S, bend; T, H-bonded turn.

**Figure 4:**
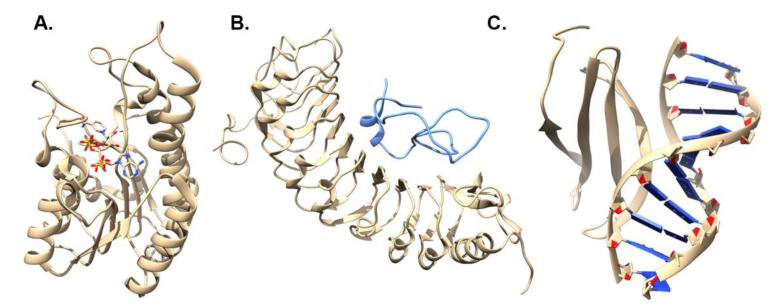
Crystal structure examples given for each feature of MSALigMap. A 3D structure is provided for visualization of the analyzed part for each given example: protein–ligand, 3WXB, carbonyl reductase, *Gallus gallus* [23] (**A**); protein–peptide, 6QWN, leucine-rich repeat (LRR) extension proteins (LRXs)/RALF, *Arabidopsis thaliana* [24] (**B**); protein–DNA, 2LEX, AtWRKY4, *Arabidopsis thaliana* [25] (**C**).

The main features of MSALigMap are the color-coded secondary structure alignment of the PDB structure and the mapping of ligand binding amino-acid residues onto non-PDB sequences (Figure 3A). Next, the tool displays the mapped amino acids that form both hydrogen-bonded and non-bonded interactions for easy comparison (Figure 3B). To exemplify the use of MSALigMap, we chose PDB crystal structures, 3WXB [23] and 3O26 [30], for two proteins (carbonyl reductase and salutaridine reductase, respectively) that are short-chain dehydrogenases bound with NADPH (Table 2, Figure 4A). We used these structures to annotate the NADPH cofactor binding amino-acid residues of the protein sequences PORA (O48741) [31], PORB (P21218) [31], and PORC (Q42536) [32], the three forms of NADPH:protochlorophyllide oxidoreductase (POR) in *Arabidopsis thaliana* (Arabidopsis) [33] (Table 2). The input file to be provided was a multiple FASTA file containing the protein sequences of the three Arabidopsis POR proteins and the two crystal structures. Secondly, information containing the PDB code with the chain ID (as provided in PDB) in a comma-separated format (3WXB:A, 3O26:A) was added.

The mapped active-site amino-acid residues are color-coded based on their physicochemical properties (Clustal X color palette); hydrogen-bonded amino acids are designated in bold, and non-bonded interacting amino acids are underlined. Secondary structure information about the PDB 3D crystal structures is provided to facilitate a comparison of the conservation of secondary structure elements across the sequences of protein crystal structures. The results of the sequence alignment of both bonded and non-bonded amino-acid residues are shown below to illustrate the level of conservation of active-site amino acids among the proteins of interest. The alignment of amino acids of the binding site alone is provided, where identical and mutated/substituted amino acids are color-shaded in red and blue, respectively. In the current analysis, of the 13 amino acids that formed a hydrogen bond between the ligand NDP and the protein, 4 were highly conserved across the sequences used in the study. However, of the 33 amino acids that were identified as forming non-bonded interactions, 9 amino acids were identified to be highly conserved.

### 3.2. MSALigMap Example: Protein–Peptide Analysis

**Figure 5:**
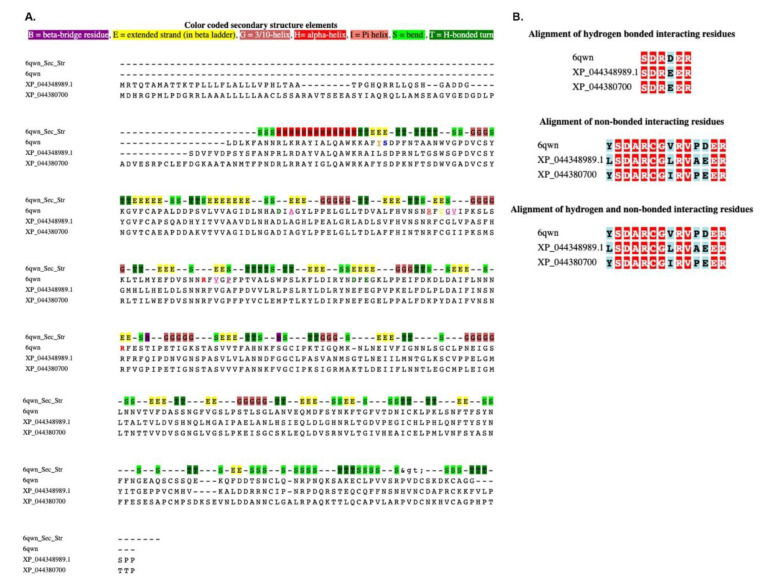
Multiple sequence alignment of leucine-rich repeat (LRR) extension proteins (LRXs) is used as a representative example of a protein–peptide analysis: (**A**) alignment with color-coded secondary structures; (**B**) hydrogen-bonded and non-bonded interacting residues retrieved with the alignment. Conserved amino acids are shown in bold/are framed with a square. B, beta-bridge residue; E, extended strand (in beta ladder); G, 3/10 helix; H, hydrogen bond; I, Pi-helix; S, bend; T, H-bonded turn.

Cell-wall-monitoring leucine-rich repeat (LRR) extension proteins (LRXs) represent an example of proteins that bind to redundant signaling RALF peptides. The protein sequence of the LRX crystal structure of the LRX protein in complex with the RALF peptide in Arabidopsis (PDB code: 6QWN) [24] was used for searching homologous sequences in *Triticum aestivum* (wheat) using the BLASTP program (Table 2, Figure 4B). Two homologous protein sequences, leucine-rich repeat extension-like protein 4 (XP_044348989) and pollen-specific leucine-rich repeat extension-like protein 4 (XP_044380700), were selected to map the RALF peptide binding sites using the structural information from homologous structural data of Arabidopsis (Table 2). The secondary structural information of 6QWN and the hydrogen-bonded and non-hydrogen-bonded interactions are shown in Figure 5A. The binding-site comparison between the LRXs of Arabidopsis and wheat revealed high conservation. Figure 5B displays the mapped hydrogen-bonded and non-bonded interactions between the LRX proteins sequences of Arabidopsis and wheat.

### 3.3. MSALigMap Example: Protein–DNA Analysis

**Figure 6:**
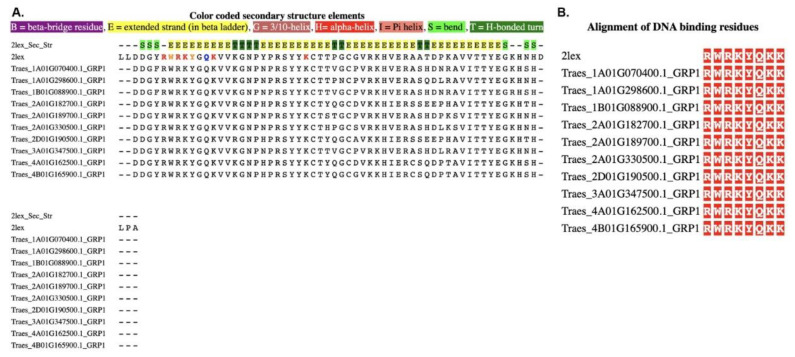
Multiple sequence alignment of WRKY transcription factor is used as a representative example of a protein–DNA analysis: (**A**) alignment with color-coded secondary structures; (**B**) alignment of the mapped residues retrieved with the alignment. Conserved amino acids are shown in bold/are framed with a square. B, beta-bridge residue; E, extended strand (in beta ladder); G, 3/10 helix; H, hydrogen bond; I, Pi-helix; S, bend; T, H-bonded turn.

The output of the protein–DNA analysis was very similar to that of the protein–ligand analysis. To illustrate the use of our tool, we chose the NMR structure of the Arabidopsis WRKY4 domain, AtWRKY4, in complex with DNA (PDB code: 2LEX) [25] (Table 2, Figure 4C). A previous study reported 297 WRKY genes in the wheat genome, of which 194 representative sequences were classified into groups I, II, and III [34]. For the current example, we used these 194 WRKY sequences to map their DNA binding sites [34]. We used this structural information of Arabidopsis WRKY4 to annotate the binding sites of 194 WRKY domain sequences identified in the *Triticum aestivum* (wheat) genome (Figure S1). In Figure 6A,B, to present the functionality of the protein–DNA binding tool of MSALigMap, we display 10 WRKY group I sequences. The secondary structural information of the crystal structure and the DNA binding amino acids of the crystal structure are highlighted (Figure 6A). The DNA binding site in 2LEX was found within the first two beta strands. Furthermore, the mapped binding sites of the crystal structure and the user-provided sequences are separately displayed to show the conservation between the sequences (Figure 6B). Comparing the 194 WRKY protein sequences of wheat with the crystal structure of Arabidopsis (PDB code: 2LEX) revealed that the DNA binding sites between these sequences are highly conserved. Three of the eight amino acids in the DNA binding site (Figure S1) that bind to the DNA molecule are highly conserved across all the sequences. Similarly, the DNA binding amino acids of 56 transcription factor families could be mapped to further understand DNA binding amino acids conservation within each family and subfamily of transcription factors. Using the current tool, the DNA binding amino acids of all transcription factor proteins in the genome of wheat could be annotated and compared for studying protein evolution in the different genes of the genome.

## 4. Discussion

It is not possible to use the tools presented here to distinguish whether mapped protein–ligand interactions are within the protein backbone or sidechain; for that purpose, the recently launched LiBiSco program can be used [35]. The ligand binding sites mapped using one or many crystal structures can be applied to several sequences and be reliable, as long as the protein sequences have similar function and sequence similarity greater than 30%, a typical cut off for protein modelling. Other very useful similar tools exist online, but they have limitations related to showing the binding sites of the sequence (e.g., XSult [20] or Alignment-Annotator web server [36]) or they have limited annotations (e.g., the SwissRegulon database of genome-wide annotations of regulatory sites currently has only 17 prokaryotes and 3 eukaryotes in their collection [37,38]. Thus, the proposed MSALigMap tool is novel and not limited in capabilities in terms of showing the binding sites in the sequence output for protein–ligand, protein–peptide, and protein–DNA complexes. MSALigMap facilitates the functional mapping of amino acids onto sequences that are obtained with whole-genome sequencing and have limited information in databases.

An important application of functional annotation is the mapping of ligand and DNA binding amino-acid residues from characterized proteins onto novel protein sequences that are generated in genome sequencing projects. In this context, MSALigMap is presented as a tool for mapping the active-site amino-acid residues that bind to either ligand or DNA onto the sequences of proteins with unknown functions by transferring information extracted from structural data of protein structural complexes. These mapped residues can be then used by users to carry out further experimental studies for exploring the efficiency of these mapped amino acids; alternatively, any in silico tools, such as Variant Effect Predictor (VEP) [39], or available machine learning approaches [40] can be used to understand the loss of function or their effect in binding efficiency. We believe that MSALigMap will be a useful tool for the functional annotation community. The analysis can also be applied to sequences from different clinical strains to map drug binding or DNA binding amino-acid residues, thus helping to identify the positions with mutations that can be further correlated to phenotypic characteristics. However, the major limitation of the server is that the submitted sequences used for mapping should have similar functions and sequence identity above the twilight zone (>30% identity). The future update of MSALigMap will possibly include options for mapping binding-site information separately for individual domain sequences of multidomain protein families.

## Figures and Tables

**Table 1:** MSALigMap alignment formatting of residue type, secondary structural features, and interaction types.

Structural Feature	Format
Alpha helix	H
Beta strand	E
310 helix	G
Pi helix	I
Bend	S
Beta–bridge	B
Turn	T
Hydrogen bond	Bold
Non-bonded interaction	Underlined
Residue type	ClustalX color palette

**Table 2:** Summary of example complexes provided for use of the different MSALigMap features.

MSALigMap	Accession No.	Name, Organism, Citation
Protein–ligand	3WXB 3O26 O48741 P21218 Q42536	carbonyl reductase, *Gallus gallus* [23] salutaridine reductase, *Papaver somniferum* [30] NADPH:protochlorophyllide oxidoreductase A (PORA), *Arabidopsis thaliana* [31] PORB, *Arabidopsis thaliana* [31] PORC, *Arabidopsis thaliana* [32]
Protein–peptide	6QWN XP_044348989 XP_044380700	leucine-rich repeat (LRR) extension proteins (LRXs)/RALF, *Arabidopsis thaliana* [24] leucine-rich repeat extension-like protein 4, *Triticum aestivum* pollen-specific leucine-rich repeat extension-like protein 4, *Triticum aestivum*
Protein–DNA	2LEX WRKY	AtWRKY4, *Arabidopsis thaliana* [25] TaWRKY, *Triticum aestivum* [34]

## Data Availability

The data that support the findings of this study are available from the corresponding author upon reasonable request.

## Supplementary Materials

The following supporting information can be downloaded at: https://www.mdpi.com/article/10.3390/life12122082/s1, Figure S1: Annotation of the DNA binding site of 194 WRKY domain sequences identified in the *Triticum aestivum* (wheat) genome.
