# Supplementary material for: MSALigMap—A Tool for Mapping Active-Site Amino Acids in PDB Structures onto Known and Novel Unannotated Homologous Sequences with Similar Function
Source: Life (Basel). 2022 Dec 12;12(12):2082. doi: 10.3390/life12122082 (PMC9784966; doi:10.3390/life12122082)
Supplement: Supplementary file 1 [file life-12-02082-s001.zip › life-2040570-supplementary.pdf]

Figure S1

Alignment of DNA binding residues

A.

2lex	R	W	R	K	Y	Q	K	K
Traes_1A01G070400.1_GRP1	R	W	R	K	Y	Q	K	K
Traes_1A01G121500.1_GRP2	K	W	R	K	Y	K	K	R
Traes_1A01G197400.1_GRP2	A	W	R	K	Y	Q	K	R
Traes_1A01G298600.1_GRP1	R	W	R	K	Y	Q	K	K
Traes_1A01G300900.1_GRP3	I	W	R	K	Y	Q	K	R
Traes_1A01G301000.1_GRP3	H	W	R	K	Y	E	K	R
Traes_1A01G301100.1_GRP3	I	W	R	K	Y	Q	K	R
Traes_1A01G301200.1_GRP3	H	W	R	K	Y	E	K	R
Traes_1A01G348600.1_GRP2	R	W	R	K	Y	Q	K	R
Traes_1A01G358100.1_GRP2	K	W	R	K	Y	K	K	R
Traes_1A01G358400.1_GRP2	K	W	R	K	Y	K	K	R
Traes_1A01G401800.1_GRP2	R	W	R	K	Y	Q	K	R
Traes_1A01G410500.1_GRP3	S	W	R	K	Y	Q	K	R
Traes_1A01G410600.1_GRP3	I	W	R	K	Y	Q	K	Q
Traes_1A01G410700.1_GRP3	S	W	R	K	Y	Q	K	R
Traes_1A01G421800.1_GRP2	R	W	R	K	Y	Q	K	R
Traes_1B01G088900.1_GRP1	R	W	R	K	Y	Q	K	K
Traes_1B01G243100.1_GRP3	T	W	R	K	Y	Q	K	R
Traes_1B01G310900.1_GRP3	H	W	R	K	Y	E	K	R
Traes_1B01G374700.1_GRP2	K	W	R	K	Y	K	K	R
Traes_1B01G434300.1_GRP2	Q	W	R	K	Y	Q	K	R
Traes_1B01G440300.1_GRP3	S	W	R	K	Y	Q	K	R
Traes_1B01G440500.1_GRP3	S	W	R	K	Y	Q	K	R
Traes_1B01G440600.1_GRP3	S	W	R	K	Y	Q	K	R
Traes_1B01G453200.1_GRP2	R	W	R	K	Y	Q	K	R
Traes_1B01G453300.1_GRP2	R	W	R	K	Y	Q	K	R
Traes_1B01G455000.1_GRP2	A	W	R	K	Y	Q	K	K
Traes_1D01G122400.1_GRP2	K	W	R	K	Y	K	K	R
Traes_1D01G300300.1_GRP3	I	W	R	K	Y	R	K	R
Traes_1D01G300400.1_GRP3	H	W	R	K	Y	E	K	R
Traes_1D01G300500.1_GRP3	I	W	R	K	Y	Q	K	R
Traes_1D01G300600.1_GRP3	H	W	R	K	Y	E	K	R

B.

2lex	R	W	R	K	Y	Q	K	K
Traes_1D01G300700.1_GRP3	I	W	R	K	Y	Q	K	R
Traes_1D01G300900.1_GRP3	H	W	R	K	Y	E	K	R
Traes_1D01G418000.1_GRP3	S	W	R	K	Y	Q	K	R
Traes_1D01G418400.1_GRP3	S	W	R	K	Y	Q	K	R
Traes_1D01G418500.1_GRP3	S	W	R	K	Y	Q	K	R
Traes_1D01G418600.1_GRP3	S	W	R	K	Y	Q	K	R
Traes_1D01G429800.1_GRP2	R	W	R	K	Y	Q	K	R
Traes_1D01G429900.1_GRP2	R	W	R	K	Y	Q	K	R
Traes_1D01G430000.1_GRP2	R	W	R	K	Y	Q	K	R
Traes_1D01G431600.1_GRP2	A	W	R	K	Y	Q	K	K
Traes_2A01G011000.1_GRP2	R	W	R	K	Y	Q	K	R
Traes_2A01G019300.1_GRP3	H	W	R	K	Y	Q	K	K
Traes_2A01G043600.1_GRP3	V	W	T	K	Y	Q	K	R
Traes_2A01G043700.1_GRP3	M	W	T	K	Y	Q	K	R
Traes_2A01G043800.1_GRP3	V	W	T	K	Y	Q	K	R
Traes_2A01G052100.1_GRP3	H	W	R	K	Y	Q	K	R
Traes_2A01G104800.1_GRP3	S	W	R	K	Y	Q	K	R
Traes_2A01G104900.1_GRP3	T	W	R	K	Y	Q	K	R
Traes_2A01G161500.1_GRP3	S	W	R	K	Y	Q	K	R
Traes_2A01G182700.1_GRP1	R	W	R	K	Y	Q	K	K
Traes_2A01G189700.1_GRP1	R	W	R	K	Y	Q	K	K
Traes_2A01G261900.1_GRP2	R	W	R	K	Y	Q	K	R
Traes_2A01G270200.1_GRP2	S	W	R	K	Y	Q	K	K
Traes_2A01G330500.1_GRP1	R	W	R	K	Y	Q	K	K
Traes_2A01G392100.1_GRP2	K	W	R	K	Y	Q	K	R
Traes_2A01G433000.1_GRP2	S	W	R	K	Y	Q	K	K
Traes_2A01G443800.1_GRP2	A	W	R	K	Y	Q	K	R
Traes_2A01G489500.1_GRP3	T	W	R	K	Y	Q	K	R
Traes_2A01G497400.1_GRP3	Q	W	R	K	Y	Q	K	K
Traes_2B01G056300.1_GRP3	V	W	T	K	Y	Q	K	R
Traes_2B01G056400.1_GRP3	M	W	T	K	Y	Q	K	R
Traes_2B01G121800.1_GRP3	S	W	R	K	Y	Q	K	R
Traes_2B01G121900.1_GRP3	T	W	R	K	Y	Q	K	R
Traes_2B01G187500.1_GRP3	S	W	R	K	Y	Q	K	R
Traes_2B01G280300.1_GRP2	R	W	R	K	Y	Q	K	R
Traes_2B01G517400.1_GRP3	T	W	R	K	Y	Q	K	R

C.

Sequence	1	2	3	4	5	6	7	8
2lex	R	W	R	K	Y	Q	K	K
Traes_2D01G042700.1_GRP3	V	W	T	K	Y	Q	K	R
Traes_2D01G051000.1_GRP3	H	W	R	K	Y	Q	K	R
Traes_2D01G104500.1_GRP3	S	W	R	K	Y	Q	K	R
Traes_2D01G104600.1_GRP3	T	W	R	K	Y	Q	K	R
Traes_2D01G168600.1_GRP3	S	W	R	K	Y	Q	K	R
Traes_2D01G190500.1_GRP1	R	W	R	K	Y	Q	K	K
Traes_2D01G262100.1_GRP2	R	W	R	K	Y	Q	K	R
Traes_2D01G489700.1_GRP3	T	W	R	K	Y	Q	K	R
Traes_2D01G497600.1_GRP3	Q	W	R	K	Y	Q	K	K
Traes_2D01G497700.1_GRP3	Q	W	R	K	Y	Q	K	K
Traes_3A01G111200.1_GRP2	R	W	R	K	Y	K	K	R
Traes_3A01G116200.1_GRP2	K	W	R	K	Y	Q	K	R
Traes_3A01G167200.1_GRP2	Q	W	R	K	Y	Q	K	R
Traes_3A01G167200.2_GRP2	Q	W	R	K	Y	Q	K	R
Traes_3A01G209800.1_GRP2	R	W	R	K	Y	K	K	R
Traes_3A01G228600.1_GRP2	R	W	R	K	Y	Q	K	R
Traes_3A01G229300.1_GRP2	A	W	R	K	Y	Q	K	K
Traes_3A01G242500.1_GRP2	R	W	R	K	Y	Q	K	R
Traes_3A01G248700.1_GRP2	K	W	R	K	Y	K	K	R
Traes_3A01G280400.1_GRP2	R	W	R	K	Y	Q	K	R
Traes_3A01G281900.1_GRP2	A	W	R	K	Y	Q	K	R
Traes_3A01G289700.1_GRP2	A	W	R	K	Y	Q	K	R
Traes_3A01G343000.1_GRP3	Q	W	R	K	Y	Q	K	R
Traes_3A01G343100.1_GRP3	L	W	R	K	Y	Q	K	R
Traes_3A01G343500.1_GRP3	K	W	K	K	Y	Q	K	R
Traes_3A01G343600.1_GRP3	Q	W	R	K	Y	Q	K	R
Traes_3A01G343700.1_GRP3	V	W	R	K	Y	Q	K	R
Traes_3A01G343900.1_GRP3	V	W	R	K	Y	Q	K	R
Traes_3A01G347500.1_GRP1	R	W	R	K	Y	Q	K	K
Traes_3A01G360900.1_GRP2	R	W	R	K	Y	Q	K	K
Traes_3A01G527300.1_GRP2	K	W	R	K	Y	Q	K	R
Traes_3B01G129900.1_GRP2	R	W	R	K	Y	K	K	R
Traes_3B01G130000.1_GRP2	Q	W	R	K	Y	Q	K	R
Traes_3B01G135300.1_GRP2	K	W	R	K	Y	Q	K	R
Traes_3B01G199000.2_GRP2	Q	W	R	K	Y	Q	K	R
Traes_3B01G240200.1_GRP2	R	W	R	K	Y	K	K	R

D.

Sequence	1	2	3	4	5	6	7	8
2lex	R	W	R	K	Y	Q	K	K
Traes_3B01G256000.1_GRP2	R	W	R	K	Y	Q	K	R
Traes_3B01G277300.1_GRP2	K	W	R	K	Y	K	K	R
Traes_3B01G375600.1_GRP3	Q	W	R	K	Y	Q	K	R
Traes_3B01G375700.1_GRP3	L	W	R	K	Y	Q	K	R
Traes_3B01G375800.1_GRP3	V	W	R	K	Y	Q	K	R
Traes_3B01G393300.1_GRP2	R	W	R	K	Y	Q	K	K
Traes_3D01G029100.1_GRP3	S	W	R	K	Y	Q	K	R
Traes_3D01G113200.1_GRP2	R	W	R	K	Y	K	K	R
Traes_3D01G238300.1_GRP3	S	W	R	K	Y	Q	K	R
Traes_3D01G248500.1_GRP2	K	W	R	K	Y	K	K	R
Traes_3D01G337100.1_GRP3	L	W	R	K	Y	Q	K	R
Traes_3D01G337300.1_GRP3	K	W	K	K	Y	Q	K	R
Traes_3D01G337400.1_GRP3	Q	W	R	K	Y	Q	K	R
Traes_3D01G337500.1_GRP3	V	W	R	K	Y	Q	K	R
Traes_3D01G337600.1_GRP3	V	W	R	K	Y	Q	K	R
Traes_4A01G128100.1_GRP3	Q	W	R	K	Y	E	K	R
Traes_4A01G135000.1_GRP3	I	W	R	K	M	R	K	–
Traes_4A01G162500.1_GRP1	R	W	R	K	Y	Q	K	K
Traes_4A01G193600.1_GRP3	Q	W	R	K	Y	E	K	R
Traes_4A01G246000.1_GRP2	S	W	R	K	Y	Q	K	K
Traes_4A01G320800.1_GRP2	A	W	R	K	Y	Q	K	R
Traes_4A01G396200.1_GRP2	Q	W	R	K	Y	Q	K	R
Traes_4A01G396300.1_GRP2	Q	W	R	K	Y	Q	K	R
Traes_4A01G396400.1_GRP2	Q	W	R	K	Y	Q	K	R
Traes_4B01G165900.1_GRP1	R	W	R	K	Y	Q	K	K
Traes_4B01G170100.1_GRP3	I	W	R	K	Y	Q	K	R
Traes_4D01G014100.1_GRP2	Q	W	K	K	Y	Q	K	R
Traes_4D01G172200.1_GRP3	I	W	R	K	Y	Q	K	R
Traes_4D01G320000.1_GRP2	A	W	R	K	Y	Q	K	K
Traes_4D01G320200.1_GRP2	A	W	R	K	Y	Q	K	K
Traes_5A01G059100.1_GRP2	S	W	R	K	Y	Q	K	K
Traes_5A01G060200.1_GRP2	S	W	R	K	Y	Q	K	R
Traes_5A01G104900.1_GRP1	R	W	R	K	Y	Q	K	K
Traes_5A01G146500.1_GRP3	Q	W	R	K	Y	Q	K	R
Traes_5A01G146700.1_GRP3	Q	W	R	K	Y	Q	K	K
Traes_5A01G185600.1_GRP3	S	W	R	K	Y	Q	K	R

E.

Sequence	1	2	3	4	5	6	7	8
2lex	R	W	R	K	Y	Q	K	K
Traes_5A01G185700.1_GRP3	S	W	R	K	Y	Q	K	R
Traes_5A01G225500.1_GRP2	Q	W	R	K	Y	Q	K	R
Traes_5A01G225600.1_GRP2	Q	W	R	K	Y	Q	K	R
Traes_5A01G258100.1_GRP1	R	W	R	K	Y	Q	K	K
Traes_5A01G344100.1_GRP3	S	W	R	K	Y	Q	K	R
Traes_5A01G373100.1_GRP2	S	W	R	K	Y	Q	K	R
Traes_5A01G381000.1_GRP2	S	W	R	K	Y	Q	K	K
Traes_5A01G395100.1_GRP2	R	W	R	K	Y	Q	K	R
Traes_5A01G396800.1_GRP1	R	W	R	K	Y	Q	K	K
Traes_5A01G443600.1_GRP2	S	W	R	K	Y	Q	K	R
Traes_5A01G495800.1_GRP2	A	W	R	K	Y	Q	K	K
Traes_5B01G066400.1_GRP2	S	W	R	K	Y	Q	K	K
Traes_5B01G145500.1_GRP3	Q	W	R	K	Y	Q	K	K
Traes_5B01G183800.1_GRP3	S	W	R	K	Y	Q	K	R
Traes_5B01G375100.1_GRP2	S	W	R	K	Y	Q	K	R
Traes_5B01G401800.1_GRP1	R	W	R	K	Y	Q	K	K
Traes_5D01G145600.1_GRP3	Q	W	R	K	Y	Q	K	K
Traes_5D01G145800.1_GRP3	Q	W	R	K	Y	Q	K	R
Traes_5D01G190800.1_GRP3	S	W	R	K	Y	Q	K	R
Traes_5D01G232800.1_GRP2	Q	W	R	K	Y	Q	K	R
Traes_5D01G349800.1_GRP3	S	W	R	K	Y	Q	K	R
Traes_5D01G349800.2_GRP3	S	W	S	K	Y	Q	K	R
Traes_5D01G451000.1_GRP2	S	W	R	K	Y	Q	K	R
Traes_6A01G080500.1_GRP3	T	W	R	K	Y	Q	K	R
Traes_6A01G146900.1_GRP2	Q	W	R	K	Y	Q	K	R
Traes_6A01G242000.1_GRP2	K	W	R	K	Y	Q	K	R
Traes_6A01G267500.1_GRP2	A	W	R	K	Y	Q	K	R
Traes_6A01G326500.1_GRP2	Q	W	R	K	Y	Q	K	R
Traes_7A01G096500.1_GRP2	Q	W	R	K	Y	Q	K	R
Traes_7A01G116000.1_GRP3	S	W	R	K	Y	Q	K	R
Traes_7A01G240800.1_GRP1	R	W	R	K	Y	Q	K	K
Traes_7A01G294800.2_GRP1	R	W	R	K	Y	Q	K	R
Traes_7A01G338500.1_GRP3	A	W	R	K	Y	Q	K	R
Traes_7A01G343000.1_GRP2	A	W	R	K	Y	Q	K	R
Traes_7A01G508700.1_GRP2	Q	W	R	K	Y	Q	K	R
Traes_7A01G508800.1_GRP2	Q	W	R	K	Y	Q	K	R

F.

Sequence	1	2	3	4	5	6	7	8
2lex	R	W	R	K	Y	Q	K	K
Traes_7B01G136400.1_GRP1	R	W	R	K	Y	Q	K	K
Traes_7B01G249700.1_GRP3	A	W	R	K	Y	Q	K	R
Traes_7B01G418400.1_GRP2	Q	W	R	K	Y	Q	K	R
Traes_7B01G418500.1_GRP2	Q	W	R	K	Y	Q	K	R
Traes_7B01G418600.1_GRP2	Q	W	R	K	Y	Q	K	R
Traes_7D01G092300.1_GRP2	Q	W	R	K	Y	Q	K	R
Traes_7D01G092400.1_GRP2	Q	W	R	K	Y	Q	K	R
Traes_7D01G092500.1_GRP2	Q	W	R	K	Y	Q	K	R
Traes_7D01G092600.1_GRP2	Q	W	R	K	Y	Q	K	R
Traes_7D01G239800.1_GRP1	R	W	R	K	Y	Q	K	K
Traes_7D01G345700.1_GRP3	A	W	R	K	Y	Q	K	R
Traes_7D01G497300.1_GRP2	Q	W	R	K	Y	Q	K	R
Traes_7D01G497400.1_GRP2	Q	W	R	K	Y	Q	K	R
Traes_7D01G497700.1_GRP2	Q	W	R	K	Y	Q	K	R
Traes_7D01G497800.1_GRP2	Q	W	R	K	Y	Q	K	R
